# The novel regulatory role of lncRNA‐miRNA‐mRNA axis in cardiovascular diseases

**DOI:** 10.1111/jcmm.13866

**Published:** 2018-09-06

**Authors:** Ying Huang

**Affiliations:** ^1^ Department of Cardiology the First Affiliated Hospital of Anhui Medical University Hefei China

**Keywords:** cardiovascular disease, long noncoding RNA, microRNA, mRNA

## Abstract

Long noncoding RNAs (lncRNAs) are RNAs longer than 200 nt in length that are characterized by low levels of sequence conservation and expression; lncRNAs modulate various biological functions at epigenetic, transcriptional and post‐transcriptional levels, or directly regulate protein activity. As a family of small and evolutionarily conserved noncoding RNAs, microRNAs (miRNAs) are capable of regulating physiological and pathological processes via inhibiting target mRNA translation or promoting mRNA degradation. A number of studies have confirmed that both lncRNAs and miRNAs are closely associated with the development of cardiovascular diseases (CVDs), such as cardiac remodelling, heart failure, myocardial injury and arrhythmia, and that they act as biomarkers, potential therapeutic targets or strong indicators of prognosis; however, the underlying molecular mechanism has not been elucidated. Recently, emerging evidence showed that the novel regulatory mechanism underlying the crosstalk among lncRNAs, miRNAs and mRNAs plays a pivotal role in the pathophysiological processes of CVDs in response to stress stimuli. In this review, I comprehensively summarized the regulatory relationship of lncRNAs, miRNAs and mRNAs and highlighted the important role of the lncRNA‐miRNA‐mRNA axis in CVDs.

## 1 INTRODUCTION

Cardiovascular disease (CVD) remains the leading cause of mortality worldwide, although approximately 25% reduction in mortality is expected by 2025, resulting in part from the development of novel diagnostic techniques and therapeutics.^1^ In response to stress stimuli, the hypertrophy, apoptosis, necrosis and autophagy of cardiomyocytes, as well as the proliferation and differentiation of cardiac fibroblasts, endothelial cells (ECs) and vascular smooth muscle cells (VSMCs), contribute to the initiation and progression of CVDs, such as cardiac hypertrophy and fibrosis, heart failure (HF), myocardial injury, atherosclerosis and arrhythmias. Unfortunately, the molecular mechanisms of these pathological and pathophysiological processes have not been completely elucidated. Seeking and modulating the key molecules involved in CVDs are essential to explore effective prevention measures and treatment methods.

Recently, a variety of studies have validated that noncoding RNAs (ncRNAs) participated in the development of diverse CVDs by regulating cell differentiation,^2^ proliferation,^3^ apoptosis,^4^ necrosis^5^ and autophagy.^6^ ncRNAs, which have been extensively investigated in the past decades, are characterized by having no significant protein‐coding potential. As the two major members of the ncRNA family, long noncoding RNAs (lncRNAs) and microRNAs (miRNAs) have been demonstrated by accumulating data to exert pivotal roles in regulating the physiological and pathophysiological processes of CVDs.^7^, ^8^ lncRNAs possess a mRNA‐like structure and a length greater that 200 nt, and some have a poly (A) tail. According to the NONCODE database (http://www.noncode.org), there are 102 783, 87 553 and 27 793 lncRNA genes for human, mouse and rat, respectively; in comparison, human have 19 836 human protein‐coding genes (Ensemble 90). Alternative splicing during differentiation causes lncRNAs to exhibit lower levels of sequence conservation and expression than protein‐coding genes, but growing evidence has confirmed that lncRNAs maintain pivotal and multiple functions in a vast array of biological processes despite their limited expression levels. To date, multiple lncRNAs have been well documented due to the development of high‐throughput RNA sequencing (RNA‐seq) techniques; they can be divided into five subclasses as sense, antisense, bidirectional, intergenic and intronic lncRNAs, based on their position in the genome or relation to the coding gene.^9^ Functional analysis showed that lncRNAs could regulate genes expression at epigenetic,^10^ transcriptional^11^ and post‐transcriptional levels,^12^ or could directly modulate protein activity.^13^ Unlike lncRNAs, miRNAs are a family of evolutionarily conserved, endogenous, ncRNAs approximately 22 nt in length, that can bind to the 3′‐untranslated region (3′‐UTR) of mRNAs through imperfect complementarity.^14^ miRNAs may regulate more than 30% of genes and play a significant role in the regulation of biological activities in cells by inhibiting target mRNA translation or promoting mRNA degradation.^15^, ^16^

It is of note that lncRNA and miRNA can modulate the initiation and progression of CVDs via regulating different molecular mechanisms. For instance, the inhibition of Meg3 suppressed cardiac fibrosis and hypertrophy by decreasing MMP‐2 expression.^17^ The up‐regulation of miR‐29 could reduce lesion size and necrotic zones via targeting Col1A and Col3A.^18^ Recently, a novel regulatory mechanism among lncRNAs, miRNAs and their mRNA targets has been annotated in CVDs by pull‐down assay, chromatin immunoprecipitation (ChIP), luciferase reporter gene assay and quantitative reverse transcription polymerase chain reaction (qRT‐PCR) methods, although a plurality of evidence has revealed that lncRNA‐miRNA‐mRNA cascades participated in various pathophysiological processes in the field of oncology.^7^, ^19^, ^20^, ^21^ In this review, four kinds of regulatory relationships were summarized: (a) the role of lncRNAs as miRNA sponges; (b) the coexpression of lncRNAs with miRNAs; (c) the reciprocal repression of lncRNAs and miRNAs; and (d) the role of miRNAs as negative regulators of lncRNAs. I made a comprehensive summary on the crosstalk among lncRNAs, miRNAs and mRNAs, and highlighted the important role of the lncRNA‐miRNA‐mRNA axis in the development of CVDs.

## 2 lncRNA AS miRNA SPONGES IN REGULATING THE BIOLOGICAL ACTIVITIES OF CARDIOMYOCYTES AND CARDIAC FIBROBLASTS

In 2014, the lncRNA AK048451, named cardiac hypertrophy related factor (CHRF), was first identified as an endogenous sponge of miR‐489 that could directly bind to miR‐489 in a sequence‐specific manner and restrain miR‐489 expression; this ability was demonstrated by analysis with the bioinformatics programme RNA hybrid, luciferase reporter gene activity assay and pull‐down assay. The enforced expression of CHRF promoted hypertrophic responses, and the inhibition of CHRF retarded cardiac hypertrophic effects induced by angiotensin II (Ang II) treatment by regulating the miR‐489 activity and the expression of its target, myeloid differentiation primary response (Myd88); these effects suggested that CHRF served as the upstream negative regulator of the miR‐489/Myd88 axis under the hypertrophic condition.^22^ Other lncRNAs have also been confirmed to act as miRNA sponge in order to modulate cardiomyocyte hypertrophy. The lncRNA growth arrest‐specific 5 (GAS5) was generally regarded as a tumour suppressor, acting as a miR‐21 sponge, that could inhibit the proliferation and promote the apoptosis of various cancer cells.^23^, ^24^ Interestingly, the study showed that GAS5 contained a binding site for miR‐23a and acted as a sponge of miR‐23a. Up‐regulated GAS5 expression inhibited cardiomyocyte hypertrophy through negatively regulating miR‐23a and its target forkhead box O3 (Foxo3a).^25^ Additionally, a previous study confirmed that myocardial infarction‐associated transcript (MIAT) possessed binding sites for miR‐150 and could serve as a miR‐150 sponge in modulating cell proliferation, apoptosis and migration.^26^ In cultured H9C2 cells, the overexpression of MIAT triggered an Ang II‐induced hypertrophic response via inhibiting the level of miR‐150 level; however, the possible target of miR‐150 was not identified in this study.^27^

Mitochondrial fission and fusion are associated with cardiomyocyte apoptosis.^28^ The lncRNA AK017121, named cardiac apoptosis‐related lncRNA (CARL), was identified as a functional sponge of miR‐539. The overexpression of CARL suppressed mitochondrial fission, apoptosis and ischaemia/reperfusion (I/R) injury by directly inhibiting the expression of miR‐539 and subsequently up‐regulating the level of its target prohibitin 2 (PHB2).^29^ The modulation of CARL‐miR‐539–PHB2 expression might provide a novel approach for the treatment of ischaemic heart disease (IHD). Another lncRNA AK009271, named mitochondrial dynamic related lncRNA (MDRL), is also involved in mitochondrial fission and fusion under stress condition. MDRL, which contains a target site for miR‐361, could directly interact with miR‐361 and suppressed the expression and activity of miR‐361, thus reducing mitochondrial fission and apoptosis upon anoxia/reoxygenation treatment. Although the specific target mRNA of miR‐361 was not discussed, a novel regulatory mechanism between miR‐361 and miR‐384 was explored. miR‐361 could directly bind to the primary transcript of miR‐484 and hampered the processing of pri‐miR‐484 into pre‐miR‐484 by Drosha in the nucleus. The overexpression of MDRL reduced the level of pri‐miR‐484 and increased the expression of pre‐miR‐484, while these effects were reversed when MDRL expression was knocked down. These data implied that different lncRNAs could suppress the mitochondrial fission and apoptosis by modulating different molecular pathways.^30^

Cardiac differentiation is essential to maintain normal heart development and function. The molecular mechanism underlying H19‐modulated cardiac differentiation was examined in P19CL6 cells, which are a functional in vitro model for exploring cardiomyocyte differentiation.^31^ During the late stage of cardiac differentiation of P19CL6 cells, miR‐19b was negatively regulated by H19, as demonstrated by luciferase activity assay and qRT‐PCR. The overexpression of H19 significantly inhibited cell proliferation and promoted cell apoptosis via modulating miR‐19b and its target sox6, while the down‐regulation of H19 reversed these effects.^32^

Cardiomyocyte autophagy is an evolutionarily conserved process in face of stress stimuli, but abnormal autophagy contributes to cell death.^33^ The lncRNA AK079427, named APF, played a crucial role in the regulation of cardiomyocyte autophagy by modulating miR‐188‐3p and its target ATG7. The knockdown of APF reduced autophagic vesicle formation and cell death in cardiomyocytes upon anoxia/reoxygenation treatment, as well as in I/R injury heart via inhibiting miR‐188‐3p expression and augmenting the level of ATG7.^34^ Unveiling the functional role of APF in cardiomyocyte autophagy is helpful for protecting against cardiac dysfunction. In an I/R injury model, compared to control groups, APF‐siRNA mice demonstrated a decreased diastolic left ventricular internal diameter (LVIDd) but increased fractional shortening (FS), indicating that APF may be viewed as a therapeutic target to preserve cardiac function.

Cardiomyocyte necrosis mainly contributes to heart failure, while the underlying mechanism is not fully explained.^35^ H19, which contains three potential miR‐103/107 binding sites, prevented cardiomyocyte necrosis via inhibiting the level of miR‐103/107 and its target Fas‐associated protein with death domain (FADD) in response to H_2_O_2_ treatment, while these effects were offset by silencing H19.^36^ Another lncRNA, named necrosis‐related factor (NRF), was closely related to necrotic death of cardiomyocytes by acting as an endogenous RNA sponge that interacted with miR‐873 in the cytoplasm. Silencing of NRF increased miR‐873 expression and decreased the levels of the miR‐873 targets receptor‐interacting serine/threonine‐protein kinase 1 (RIPK1) and RIPK3, which led to a sharp reduction in myocardial necrosis.^37^ Strikingly, NRF could also be regulated at the transcriptional level, and a binding site of p53 was detected in the promoter region of NRF. H_2_O_2_ treatment exacerbated the association of p53 with the NRF promoter, as determined by ChIP assay, indicating that p53 promoted the activity of NRF in response to stress. A functional assay showed that silencing p53 reduced necrotic cell death and inhibited NRF promoter activity upon H_2_O_2_ treatment, and the increased expression of miR‐873 and decreased level of RIPK1/RIPK3 were also induced by the down‐regulated P53 level.^37^ Taken together, elucidating the regulatory relationship in the lncRNA‐miRNA‐mRNA axis in the process of cardiomyocyte necrosis shed light on exploring potential therapeutic target for cardiac dysfunction.

The activation of cardiac fibroblast was also involved in the altered expression of lncRNA. In transforming growth factor (TGF)‐ß1‐activated cardiac fibroblasts, the level of GAS5 was down‐regulated, while the level of miR‐21 was up‐regulated. The overexpression of GAS5 prevented the growth of cardiac fibroblasts and inhibited the expressions of Col1A1 and α‐SMA at both the mRNA and protein levels via negatively regulating miR‐21 and its target, phosphatase and tensin homologue (PTEN). The differentiation and proliferation for cardiac fibroblasts is the main reason of cardiac fibrosis. Hence, these findings may indicate that the modulation of miR‐21/PTEN by GAS5 plays an important role in the development of cardiac fibrosis.^38^

## 3 COEXPRESSION OF lncRNAs WITH miRNA IN MODULATING THE BIOLOGICAL FUNCTIONS OF VSMCs, MACROPHAGES AND CARDIOMYOCYTES

In contrast to the lncRNAs with the ability to negatively regulate miRNAs by acting as miRNA sponges, other lncRNAs may positively regulate the level of miRNAs; this ability renovated our understanding of the relationship between lncRNAs and miRNAs. Recent studies have confirmed that the positively regulatory relationship between lncRNA and miRNA is associated with the proliferation and hypertrophy of VSMCs, which contributes to the formation of atherosclerosis. The lncRNA Ang 362 was found to be proximal to miR‐221/222 and was co‐transcribed with miR‐221/222 in VSMCs. The levels of Ang 362 and miR‐221/222 were increased by Ang II treatment at a time‐dependent manner. The down‐regulated of Ang 362 reduced the expression of miR‐221/222 and Mcm7, as well as prevented the proliferation of VSMCs, thus indicating that lncRNA Ang 362 aggravated Ang II‐triggered vascular dysfunction by positively regulating miR‐221/222.^39^ Mcm7 is associated with the initiation of DNA replication and cell cycle progression,^40^ while it remains unclear whether Mcm7 is a potential target of miR‐211/222.^39^

The lncRNA RP5‐833A20.1 is located in intron 2 of the nuclear factor IA (NFIA) sequence, and its transcription direction is opposite that of NFIA. Both the mRNA and protein levels of NFIA were negatively modulated by lncRNA RP5‐833A20.1. Interestingly, NFIA was a target of miR‐382‐5p, and down‐regulated NFIA protein expression that was induced by the overexpression of RP5‐833A20.1, which was crucial in aggravating inflammatory responses and lipid accumulation in THP‐1 macrophages, was almost totally retarded by hsa‐miR‐382‐5p inhibitors.^41^ However, the gene correlations between RP5‐833A20.1 and miR‐382‐5p remains scantly explored and needed to be further deciphered.

It has been gradually demonstrated that H19 may modulate a variety of cell biological functions by sequestering different miRNAs; H19 can serve as not only as a sponge of miRNAs, such as let‐7,^42^ miR‐103/107^36^ and miR‐19b,^32^ in cell differentiation, necrosis and proliferation, but also as a precursor of miRNAs in the regulation of cardiomyocyte hypertrophy. The miR‐675 was validated to be located in the first exon of H19,^43^ and both H19 and miR‐675 were up‐regulated in hypertrophic heart tissue. The overexpression of H19 up‐regulated miR‐675 expression and inhibited the levels of foetal genes in cardiomyocytes, while down‐regulated H19 could reverse these effects. Ca/calmodulin‐dependent protein kinase II d (CaMKIId) was a target of miR‐675, and the inhibition of CaMKIId partially overcame the enhanced cardiomyocyte hypertrophy resulting from H19 down‐regulation. Hitherto, H19 inhibited the cardiomyocyte hypertrophic effects either by positively regulating miR‐675‐CaMKIId or by directly inhibiting CaMKIId expression.^44^

Another interesting study was reported that H19 and its encoded miR‐675 were also played an important role in the control of cellular senescence, which uncovered a novel mechanism by which the same H19 and miR‐675 complex could modulate different biological functions in cells via regulating different targets. In response to stress conditions, cardiac progenitor cells (CPCs) lost their ability for the proliferation and their differentiation potential due to cellular senescence, which is an important reason leading to cardiac dysfunction. The levels of H19 and miR‐675 were significantly reduced in H_2_O_2_‐treated CPCs; this reduction could be reversed by melatonin, which had the ability to inhibit the premature senescence of CPCs in response to oxidative stress. Silencing H19 or miR‐675 obviously abolished the protection of CPCs ageing by melatonin via inhibiting the target ubiquitin‐specific protease 10 (USP10).^45^

## 4 RECIPROCAL REPRESSIONS OF lncRNAs AND miRNAs IN THE FUNCTIONAL ROLES OF EC AND CARDIAC CELLS

As a miRNA sponge, lncRNAs are capable of negatively regulating the expression of miRNA by sequence‐specific binding; on the contrary, miRNAs can also negatively modulate lncRNA expression. A recent study revealed this reciprocal repression between MALAT1 and miR‐22. The direct binding sites of miR‐22‐3p and MALAT1 were confirmed by luciferase assay. A functional study validated that miR‐22 mimics inhibited MALAT1 expression but that a miR‐22 inhibitor increased the level of MALAT1, as evaluated by qRT‐PCR; furthermore, down‐regulated MALAT1 increased miR‐22 expression in cultured ECs. The reciprocal repression between MALAT1 and miR‐22‐3p provided a new clue for the understanding of the relationship between lncRNA and miRNA. Up‐regulated miR‐22‐3p potentiated the apoptotic rate in ECs via inhibiting the target CXCR2, while the decreased percentage of apoptotic cells resulting from the down‐regulation of miR‐22‐3p was reversed by silencing MALAT1, thus implying that MALAT1 protects endothelial function partly through the miR‐22‐3p/CXCR2 pathway.^46^ Interestingly, AKT was also identified as another target gene of miR‐22‐3p, and silencing MALAT1 induced a reduction in AKT expression, but this effect was abolished by the down‐regulation of miR‐22‐3p. It is easy to find a reciprocal loop connecting MALAT1, miR‐22 and CXCR2/AKT in regulating ECs function, and this crosstalk is highly indicative of the complicated and multiple role of the lncRNA‐miRNA–mRNA cascade in CVDs.^46^

The lncRNA ROR serves as a sponge of several miRNA sequences, including miR‐145, miR‐205 and the let‐7 family, in regulating the differentiation of embryonic stem cells^47^ and the proliferation, migration and invasion of cancer cells.^48^, ^49^ The reciprocal repression mechanism between ROR and miR‐133 was found in hypertrophic cardiomyocytes. Down‐regulated ROR increased the expression of miR‐133, and miR‐133 mimics successfully inhibited the expression of ROR analysed by qRT‐PCR. Although ROR and miR‐133 might be targeted for the development of novel antihypertrophic therapeutics based on the results of previous studies, the precise gene correlations of ROR and miR‐133 have not been elucidated.^50^ It has been verified that miR‐133 could prevent cardiac hypertrophy via inhibiting its targets RhoA and Cdc42. However, it is unclear whether these mRNAs also act as effect targets of miR‐133 or are directly regulated by ROR in this situation.^51^

Atrial fibrillation (AF), which is a result of abnormal electrical activities in atrial tissues, is the characteristic of electrical remodelling contributing to cardiac failure.^52^ LncRNAs and miRNAs could modulate the progression of AF via targeting diverse mRNAs. For instance, the lncRNA AK055347 contributed to the pathogenesis of AF via the regulation of Cyp450, ATP synthase and MSS51.^53^ Up‐regulated miR‐208b abolished the expression and function of CACNA1C, CACNB2 and SERCA2 during atrial remodelling.^54^ lncRNA could mediate electrical remodelling during AF through interacting with miRNA and its target mRNA. AF was induced in three of five rabbits in the TCONS_00075467 knockdown group, and silencing of TCONS_00075467 could markedly shorten the atrial effective refractory period (AERP), action potential duration (APD), and L‐type calcium current (*I*CaL) density of primary atrial myocytes, suggesting that inhibiting the level of TCONS_00075467 was associated with the development of AF.^55^

As a lincRNA, TCONS_00075467 may act in *cis* to regulate the expression of neighbouring protein‐coding genes or may modulate target genes through *trans*‐mechanism, but the location of TCONS_00075467 is far from the upstream or downstream sequences of the known protein‐coding transcripts, and in addition, nearby genes have little relation to AF. To explore the molecular mechanisms of TCONS_00075467 in the development of AF, the altered expression of both miR‐328 and its target CACNA1C were further studied. Four potential binding sites of miR‐328 in the TCONS_00075467 sequence were identified by bioinformatics analysis. The expression of miR‐328 was up‐regulated and the protein level of CACNA1C was down‐regulated when TCONS_00075467 was knockout in vivo and in vitro, whereas miR‐328 also had the ability to negatively modulate TCONS_00075467. Uncovering the regulatory relationship among TCONS_00075467, miR‐328 and CACNA1C in the process of electrical remodelling shed new light on the understanding of AF.^55^

## 5 miRNA ACTING AS A NEGATIVE REGULATOR OF lncRNAs TO PREVENT THE PROLIFERATION AND MIGRATION OF ECs

In previous studies, MIAT was identified as a sponge of miR‐150 in regulating the biological activities of epithelial cells and myocyte cells.^24^, ^25^ Conversely, miR‐150 could negatively modulate the expression of MIAT to suppress the proliferation, migration and tube formation of ECs. Vascular endothelial factor (VEGF) was a target of miR‐150‐5p, but its level was up‐regulated when MIAT was overexpressed, implying that an interplay among MIAT, miR‐150‐5p and VEGF is crucial to modulate the microvascular function.^56^

As a lincRNA, retinal noncoding RNA3 (RNCR3) was supposed to act as competing endogenous RNAs by suppressing target miRNA level, and to ultimately further influenced mRNA expression. Nevertheless, miR‐185‐5p was identified as a negative regulator of RNCR3 in the proliferation of ECs and VSMCs. The level of RNCR3 was up‐regulated in aortic atherosclerotic lesions, and the knockdown of RNCR3 decreased the proliferation of ECs and VSMCs in the thoracic aorta. The overexpression of miR‐185‐5p reduced the expression of RNCR3, and silencing miR‐185‐5p potentiated the viability and proliferation of HUVECs, while this effect was partially reversed by RNCR3 knockdown.^57^ Krüppel‐like factor 2 (KLF2) was a target of miR‐185‐5p that was also regulated by RNCR3. Down‐regulated RNCR3 reduced the KLF2 level and suppressed the viability and proliferation of HUVECs, whereas the overexpression of KLF2 eliminated these functions. These findings suggested that the crosstalk mechanism among miR‐185, KLF2 and RNCR3 played a key role in preventing the proliferation of ECs, but more evidence is necessary to identify whether there is a reciprocal repression between miR‐185 and RNCR3 as mentioned before**.** 57

## 6 CONCLUSION AND PERSPECTIVE

**Figure 1:**
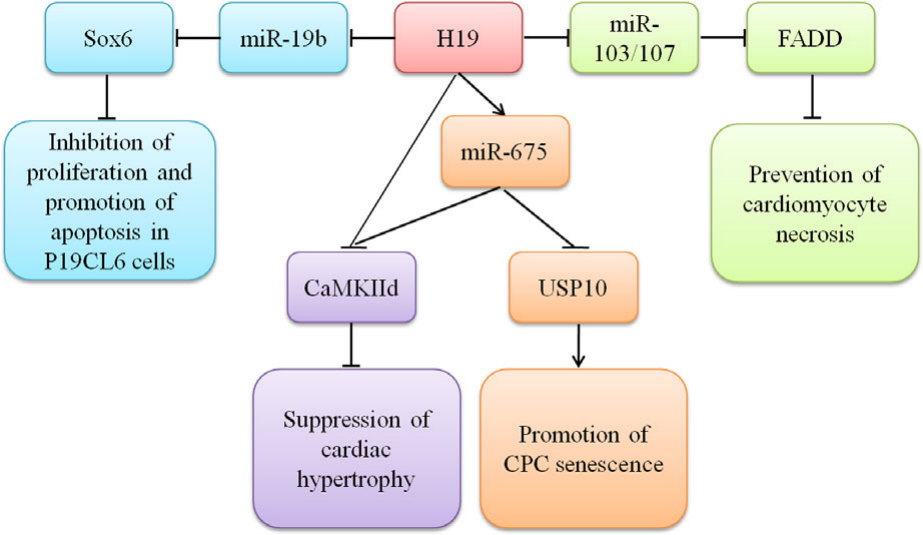
H19 as a crucial modulator in the regulation of diverse pathological processes. H19 suppressed miR‐103/107‐FADD and miR‐19b‐sox6 cascades resulting in preventing cardiomyocyte necrosis, inhibiting proliferation and promoting apoptosis in P19CL6 cells. H19 coexpression with miR‐675 attenuated cardiac hypertrophy and potentiated CPC senescence through inhibiting CaMKIId and USP10 expressions

Existing evidence suggests that ncRNAs exhibit distinctive functions in a diverse array of gene regulatory processes; however, it is still in infancy, and it is necessary to develop new approaches for the discovery and annotation of the diverse characteristics of the lncRNA‐miRNA‐mRNA axis. First, in response to different stimuli, the same lncRNA‐miRNA pair may mediate various biological processes via targeting different mRNAs. For instance, the combination of H19 and miR‐675 regulated cardiac hypertrophy and CPC senescence through inhibiting the expression of CaMKIId and USP10 (Figure 1). The CHRF‐miR‐489 played a key role in potentiating cardiac hypertrophy via targeting Myd88, and CHRF‐down‐regulated miR‐489 promoted the metastasis and epithelial‐mesenchymal transition of colorectal cancer cells via inhibiting TWIST1 expression.^58^ Second, the same lncRNA‐miRNA‐mRNA axis may modulate diverse biological functions in different diseases. The GAS5‐miR‐21‐PTEN axis suppressed breast cancer cell proliferation^23^ and increased the chemo‐sensitivity of nonsmall cell lung cancer cells to cisplatin.^59^ The CHRF‐miR‐489‐Myd88 axis was also capable of triggering the development of pulmonary fibrosis.^60^ Third, lncRNAs, miRNAs and mRNAs can be influenced by other molecules in response to stress conditions, which makes it difficult to fully explore the intrinsic regulatory mechanisms. Finally, many studies have shown that the altered expression of lncRNAs and miRNAs has identified these lncRNAs and miRNAs as potential therapeutic targets or biomarkers in the progression of CVDs.^61^, ^62^ However, owing to the lack of large‐scale clinical studies, little is known about whether the dynamic regulation of the lncRNA‐miRNA‐mRNA axis could be viewed as the effect of drug therapy or as the marker of disease development. Collectively, the crosstalk among lncRNAs, miRNAs and mRNAs seems to be complex. I have summarized four kinds of regulatory relationships among these players under stress conditions (Table 1); however, the journey of exploring the function and mechanism of the lncRNA‐miRNA‐mRNA axis in the regulation of cardiovascular physiology and pathology remains long.

**Table 1:** The lncRNA‐miRNA‐mRNA axis in CVDs

Relationships	lncRNAs	miRNAs	mRNAs	Functions	References
lncRNAs negatively regulate miRNAs	CHRF	miR‐489	Myd88	Promoted cardiac hypertrophy and dysfunction	22
	GAS5	miR‐23a	Foxo3a	Inhibited cardiac hypertrophy	25
		miR‐21	PTEN	Hampered activation of cardiac fibroblasts	38
	MIAT	miR‐150		Promoted cardiac hypertrophy	27
	CARL	miR‐539	PHB2	Inhibited mitochondrial fission, apoptosis and reduced I/R injury	29
	MDRL	miR‐361/‐484		Suppressed I/R injury and protected cardiac function	30
	H19	miR‐19b	Sox6	Inhibited proliferation and promoted apoptosis in cardiomyocytes	32
		miR‐103/‐107	FADD	Prevented I/R injury and protected cardiac function	36
	APF	miR‐188	ATG7	Suppressed I/R injury and protected cardiac function as it was down‐regulated	34
	NRF	miR‐873	RIPK1/RIPK3	Induced I/R injury and cardiac dysfunction	37
lncRNAs positively regulate miRNAs	Ang 262	miR‐221/‐222		Promoted proliferation of VSMCs	39
	RP5‐833A20.1	miR‐382‐5p	NFIA	Induced atherosclerosis	41
	H19	miR‐675	CaMKIId	Inhibited cardiac hypertrophy	44
			USP10	Protected CPCs senescence	45
Reciprocal correlations between lncRNAs and miRNAs	MALAT1	miR‐22	CXCR2/AKT	Prevented ECs apoptosis	46
	ROR	miR‐133		Promoted cardiac hypertrophy	50
	TCONS_00075467	miR‐328	CACNA1C	Induced AF as it was knockdown	55
miRNA negatively regulates lncRNA	miR‐150	MIAT	VEGF	Inhibited proliferation, migration and tube formation of ECs	56
	miR‐185	RNCR3	KLF2	Prevented proliferation of ECs	57

## CONFLICT OF INTEREST

The author confirms that this article content has no conflict of interest.
